# LC3-Associated Phagocytosis in Bacterial Infection

**DOI:** 10.3390/pathogens11080863

**Published:** 2022-07-30

**Authors:** Jin Yuan, Qiuyu Zhang, Shihua Chen, Min Yan, Lei Yue

**Affiliations:** 1 Department of Pathogen Biology and Immunology, Faculty of Basic Medical Science, Kunming Medical University, Kunming 650500, China; yuan9710042022@163.com (J.Y.); sarahkds28@hotmail.com (Q.Z.); chenshihua96@163.com (S.C.); 2 The Institute of Medical Biology, Chinese Academy of Medical Sciences and Peking Union Medical College, Kunming 650118, China

**Keywords:** LC3-associated phagocytosis, bacterial infection, phagocyte, immune evasion

## Abstract

LC3-associated phagocytosis (LAP) is a noncanonical autophagy process reported in recent years and is one of the effective mechanisms of host defense against bacterial infection. During LAP, bacteria are recognized by pattern recognition receptors (PRRs), enter the body, and then recruit LC3 onto a single-membrane phagosome to form a LAPosome. LC3 conjugation can promote the fusion of the LAPosomes with lysosomes, resulting in their maturation into phagolysosomes, which can effectively kill the identified pathogens. However, to survive in host cells, bacteria have also evolved strategies to evade killing by LAP. In this review, we summarized the mechanism of LAP in resistance to bacterial infection and the ways in which bacteria escape LAP. We aim to provide new clues for developing novel therapeutic strategies for bacterial infectious diseases.

## 1. Introduction

**Figure 1:**
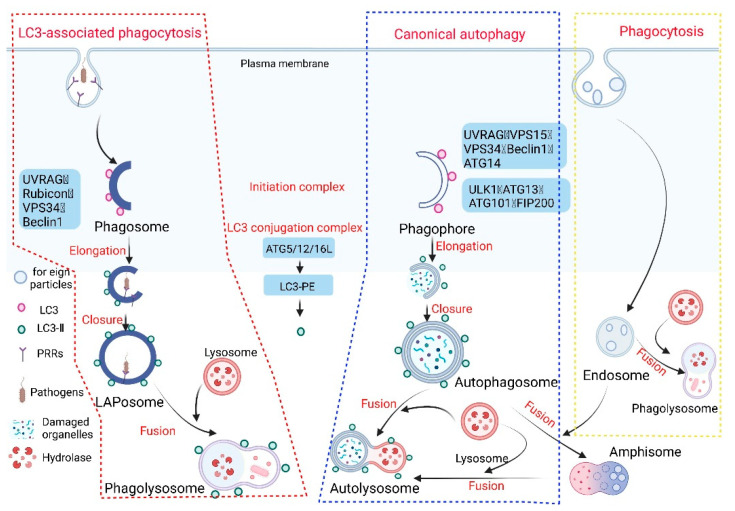
Phagocytosis, autophagy, and LC3-associated phagocytosis. Given the different origins of phagosome and autophagosome membranes, there are differences in the molecular mechanisms of LAP and autophagy. When foreign substances (e.g., apoptotic cells and invading pathogens) invade the host cell, specific receptors (such as Toll-like receptors, Fcγ receptors, or dectin-1) on the surface of the host phagocytes are activated to initiate LAP. The phagosome enables invading pathogens to be phagocytosed by the plasma membrane to form a single-membrane structure. Intracellular macromolecular proteins and damaged organelles are mainly surrounded by a double-membrane phagophore through autophagy, and then the phagosome and phagophore gradually extend and wrap around part of the cytoplasm and the organelles and proteins that need to be degraded in the cell to form the autophagosome and LAPosome. The autophagosome and endosome form an autophagosome (amphisome), and finally fuse with the lysosome to form the autophagolysosome and phagolysosome, respectively. A series of acid hydrolases are involved in the degradation of cytoplasmic substances to achieve cell homeostasis and organelle renewal (the red wireframe content represents the process of LAP occurrence, the blue wireframe content is the autophagy process, and the yellow wireframe content is the phagocytosis process).

The innate immune system is the first line of defense against the invasion of pathogenic microorganisms in the host. When they invade, the body can engulf, hydrolyze, and clear them, and manufacture the corresponding epitopes for activating the body’s immune response to the infection [1]. LC3-associated phagocytosis (LAP) plays a very important role in clearing pathogenic microbial infections, and its mechanism is different from phagocytosis and canonical autophagy (hereafter autophagy) (Figure 1).

Phagocytosis refers to the act of phagocytic cells ingesting solid particles from the surrounding environment in the form of protruding pseudopodia wraps. It is generally believed that the mechanism of this effect is the same as that of pinocytosis by the invagination of the cell membrane for the uptake of fluid or small molecules [2]. When the solid matter is adsorbed on the cell membrane, the membrane protrudes or sinks. Once the cell membranes on both sides are fused, the solid matter surrounded by the membrane is encapsulated in the cell. Phagocytosis in the body’s immune system is generally completed by professional phagocytes, which include dendritic cells, neutrophils, macrophages, and eosinophils [3]. These professional phagocytes activate phagocytosis of mammalian immune cells through attachment to pathogen-associated molecular patterns (PAMPs), which can lead to NF-κB activation. Opsonins such as C3b and antibodies can act as attachment sites, thereby immobilizing on the surface of phagocytes, and then promoting the internalization (uptake) of phagosomes through actin and myosin contraction systems, thereby forming phagosomes, which in turn ingest substances. The phagosomes fuse with lysosomes to form phagolysosomes and cause phagosome degradation.

Autophagy (referring to macroautophagy) is a process in which cells degrade their own proteins, organelles, or other intracellular components, and realize the reuse of degradation products and the renewal of organelles. Autophagy is dependent on the formation, maturation, and subcellular relocation of autophagosomes, ultimately leading to the fusion of autophagosomes with lysosomes. Autophagy can be divided into six stages: (1) pre-initiation, (2) autophagy initiation, (3) membrane vesicle extension, (4) autophagosome formation, (5) lysosome fusion, and (6) degradation and reuse. The molecular mechanism of autophagy involving multiple conserved autophagy-related proteins (ATG) has been widely described [4]. When the cell receives an autophagy-inducing signal, a membrane structure-like “liposome” is formed in the cytoplasm, and then continuously expands, forming a double-membrane structure under the electron microscope, which is called a phagophore. The phagophore will continue to extend until the components in the cytoplasm (e.g., misfolded proteins, damaged mitochondria) are surrounded, and an autophagosome is formed [5]. In this process, two ubiquitin-like coupling pathways are required, and then the fusion of autophagosome and lysosome will form an “autophagolysosome” or autolysosome, at which time the “cargo” endocytosed by the autophagosome will be degraded [6].

**Figure 2:**
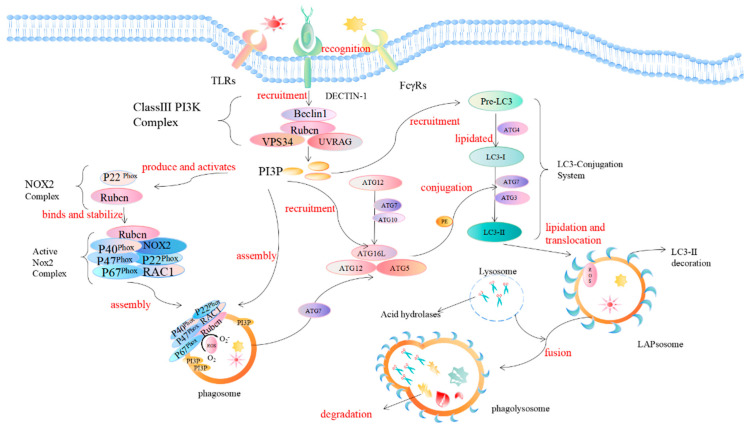
Mechanism of LC3-associated phagocytosis. During pathogen invasion, phagocyte surface-specific receptors (such as TLRs, FcγR, dectin-1) interact with PAMPs and activate the LAP pathway. Rubicon is recruited during LAP and promotes the activity of UVRAG-containing class III PI3K complexes. The PI3K complex, which is the first protein involved in LAP regulation, consists of Beclin-1, VPS34, UVRAG, and Rubicon. Rubicon maintains the stability of the PI3K complex, which in turn sustains the production of phosphatidylinositol 3-phosphate (PI3P) on the phagosome membrane, and PI3P acts as a downstream recruitment signal for autophagy. This process is necessary to stabilize the NOX2 complex, thereby maintaining the production of reactive oxygen species. PI3P is also critical for the complementation of components (such as ATG5, ATG3, ATG12, ATG7, and ATG16L) of the two subsequent ubiquitin-like conjugation systems (ATG5-ATG12-ATG16L, and the LC3-PE conjugation system) and the stabilization of the NOX2 complex to generate reactive oxygen species. The activation of the NADPH oxidase complex triggers the production of ROS on the phagosome. The production of ROS will lead to the rapid lipidation of LC3 in the phagosome membrane to form the LC3-associated phagosome (LAPosome), which is fused with the lysosome, and a series of acid hydrolases will participate in the degradation of cytoplasmic substances.

LAP is a noncanonical autophagy process reported in recent years in which microtubule-associated protein 1 light chain 3-β (LC3) binds to the phagosomal membrane using part of the autophagy mechanism [7]. Compared with autophagy that uptakes the cytoplasmic cargo, LAP is reported to target extracellular cargo. When pathogens invade the body initially, receptors on the surface of phagocytes, such as Toll-like receptors (TLRs), dendritic cell-associated C-type lectin-1 (dectin-1), and Fcγ receptors (FcγRs), recognize and interact with the PAMPs of pathogens to activate the LAP pathway [8,9,10,11,12]. The recruitment and assembly of the NADPH oxidase 2 (NOX2) complex on the initial phagosomes after LAP activation [13] is caused by the signaling cascade reaction of spleen tyrosine kinase (Syk) and protein kinase C (PKC), and is stabilized by the Rubicon protein. As part of the autophagy complex containing Beclin-1-vps34, Rubicon is a Beclin1-interacting protein and is essential for LAP maturation [14]. The recruitment of NADPH oxidase triggers the production of reactive oxygen species (ROS). ROS production leads to the rapid lipidation of LC3 and conjugation to the single-membrane phagosome which is the most different from a double-membrane vesicle in autophagy, thereby forming a vesicle decorated by LC3, which is called a LAPosome. Fusion of the LAPosomes with lysosomes results in their maturation into phagolysosomes, which can effectively eliminate engulfed pathogens, thereby improving the efficiency of phagocytosis and the killing of pathogens by phagolysosome complexes [15,16,17] (Figure 2).

LAP is a unique pathway that links signaling during phagocytosis to the recruitment of certain components of the autophagic machinery. The regulation mechanism of autophagosome formation is an important part of autophagy research. LAP and autophagy are similar, but different. In the initiation process, autophagy begins with the formation of a double-membrane vesicle (phagophore) in the cytoplasm. In this process, two protein complexes are involved in autophagy: one containing the VPS34 complex (UVRAG, Beclin1, ATG14, VPS34, VPS15) and one containing the serine/threonine kinase ULK1 complex (ULK1, FIP200, ATG13, ATG101). LAP, on the other hand, involves the formation of a single-membrane phagosome, which involves initiation complexes including UVRAG, Beclin1, and VPS34, as well as a unique regulator, Rubicon. Among them, the Rubicon molecule acts like a switch, and although Rubicon inhibits VPS34 activity during autophagy, it is required for VPS34 activity on LAPosomes [18].

NADPH oxidase 2 (NOX2/gp91phox) was first discovered in phagocytes. NADPH oxidase consists of the common integral membrane protein subunit p22^phox^, the catalytic subunit gp91^phox^, the regulatory subunits p47^phox^, p40^phox^, and p67^phox^, and the small GTPase Rac. The gp91^phox^ and p22^phox^ subunits are mainly located on the plasma membrane [19]. The C-terminus of p22^phox^ has a proline-rich region. The production of ROS requires the transfer of several other subunits (p47^phox^, p40^phox^, p67^phox^, and Rac) in the cytoplasm to the plasma membrane, following binding to gp91^phox^ and p22^phox^. During LAP, Rubicon directly interacts with the p22^phox^ subunit of NOX2 through its serine-rich region (amino acids 567–625) to stabilize the NOX2 complex for optimal and sustained ROS production, in which the NOX2 subunit p40^phox^ binding to PI3P is also the result of the action of Rubicon [20]. Therefore, the stabilization of NOX2 and the generation of ROS are very important for LC3 lipidation during the subsequent LAP process and are also required for LAP formation [21]. LC3 plays a key role in the elongation and maturation of the autophagosome membrane. LC3 lipidation requires two ubiquitin-like conjugation systems, including the ATG5-ATG12 conjugation system and the LC3-phosphatidylethanolamine (PE) conjugation system on the surface of the phagosome. The first ubiquitin-like conjugation system activates ATG12 for ATG7, which can be linked to ATG10. ATG12 binds to ATG5 and further binds to ATG16L1 to form a multimeric ATG5–ATG12–ATG16L1 complex. The second ubiquitin-like conjugation system is the cleavage of cytoplasmic LC3 by ATG4 to generate LC3-I, which is activated by ATG7, and then covalently linked to PE on the membrane surface to convert LC3-I to lipidated LC3-II. The processed LC3-II is a marker for phagophores and phagosomes to mature into autophagosomes and LAPosomes, respectively. It is also a necessary condition for the autophagosome and LAPosome to fuse with the lysosome and degrade pathogens after fusion with the lysosome. While both autophagy and LAP are characterized by LC3-II binding to membranes, LC3-II is recruited to different types of membranes, which is one of the most striking ultrastructural differences that distinguish LAPosomes from autophagosomes. Here, we only briefly describe LAP and autophagy. Although LAP and autophagy share many of the same ATGs, there are still differences in the complexes necessary for their respective processes [22,23].

Since the discovery of LAP [9], with respect to microbial infections, the list of pathogens (bacteria, virus, fungi, parasites) targeted by LAP and the knowledge about microbial evasion strategies for LAP has grown recently [24]. Different pathogenic microorganisms have different ways of responding to the killing of LAP. However, among the reports on the interaction of LAP with pathogenic microorganisms, the mechanism of bacterial infection is the most studied. Here, we highlight recent work on the LAP pathway and the mechanisms by which bacteria undermine LAP.

## 2. Biological Functions of LAP

During bacterial infection, host cells engulf invading bacteria through a single-membrane vesicle called a “phagosome”. LC3 is recruited to the bacteria-containing phagosomal membrane to form an LC3-modified vesicle called a LAPosome. The LAPosome fuses with lysosomes to degrade bacteria. LAP begins with phagocytosis, transports captured pathogens to lysosomes for degradation, and enhances pathogen-killing efficiency through the LAPosome. LAP enhances innate immune cells’ ability to kill bacteria. LAP can transport bacteria in phagocytic vesicles to transmembrane pattern recognition receptors (PRRs) oriented toward the phagosomal lumen or cytosolic PRRs located within the host-cell cytosol for more efficient recognition and killing [25]. In addition, LAP promotes phagosome–lysosome fusion, enhances the degradation of bacteria, and plays an immunomodulatory role in antimicrobial immunity [26,27,28]. To survive in host cells, bacteria have also evolved strategies to evade targeted killing and degradation by LAP. In this review, we summarize the research on the antibacterial mechanism of LAP in bacterial infections and bacterial immune escape in recent years.

## 3. LAP and Bacterial Infection

**Figure 3:**
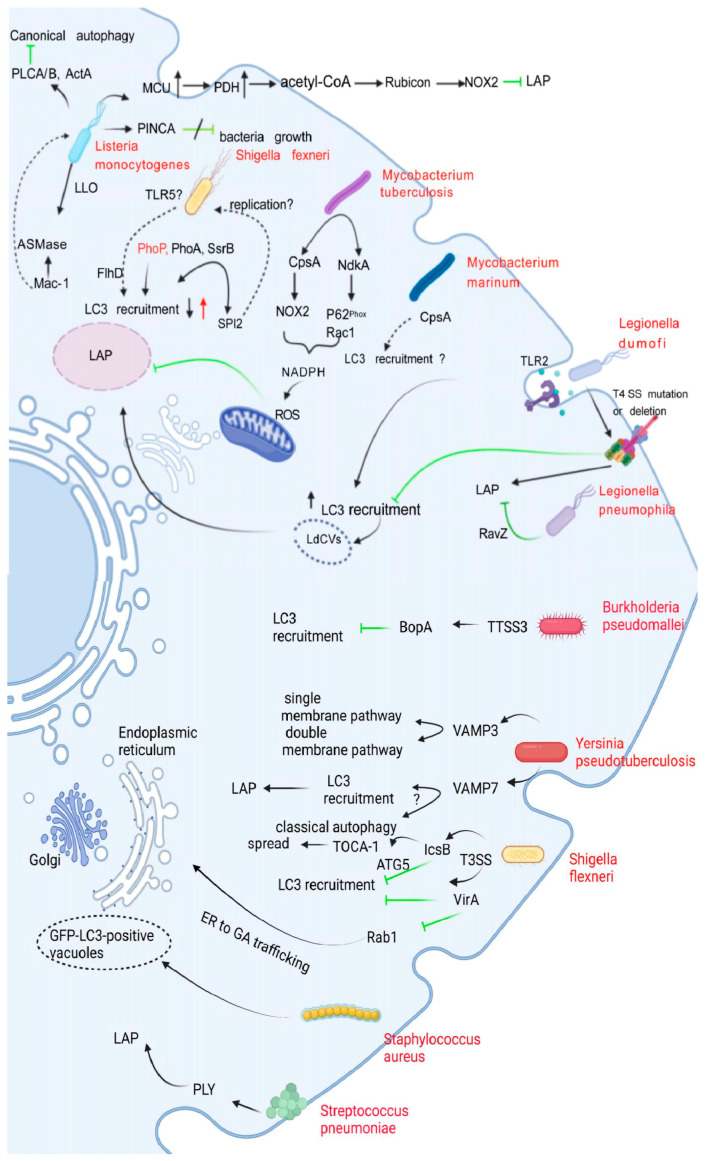
Interactions of bacteria with LAP. This figure shows the correlation between several bacteria and LAP (black arrows). Green arrows indicate inhibition. Dotted arrows represent interactions that are not yet confirmed. *L. monocytogenes* uses its own virulence factors, PLCA/B and ActA, to protect it from being recognized, killed, and degraded by autophagy targets. PINCA did not play a substantial role in anti-*L. monocytogenes* and did not inhibit bacterial growth. *L. monocytogenes* promotes the uptake of mtCa^2+^ by regulating MCU to enhance PDH activity, thereby inducing the production of acetyl-CoA. Acetyl-CoA acetylates Rubicon, resulting in decreased Rubicon content and inhibiting the interaction of Rubicon with the NOX2 complex, thereby inhibiting the formation of LAP. The interaction of *L. monocytogenes* with Mac-1 induces ASMase-mediated changes in membrane lipid composition and converts sphingomyelin to ceramide and phosphorylated choline. The deletion of the PhoP and PurA virulence factors in *S. typhimurium* increased and decreased LC3 recruitment, respectively, and SsrB is part of the bacterial regulatory system that controls the expression of SPI2 effector molecules and is required for the maintenance of *Salmonella*-containing vacuoles (SCV). However, Rubicon knockout did not affect the survival of mutant SsrB strains, possibly because SPI2 is related to bacterial replication in vivo. CpsA acts upstream of NOX2 by blocking the recruitment of NOX2 to the phagosome; *M. tuberculosis* also secretes a virulence factor called NdkA. The presence of NdkA reduces the uptake of p67^phox^ and Rac1 to the phagosome and interferes with NADPH oxidation. The enzyme complex generates ROS and may destroy LAP. LAP binds LC3 to LdCVs in a Dot/Icm T4SS-dependent manner, leading to bacterial degradation, a process that requires TLR2. *L. pneumophila* can irreversibly uncouple the conjugation function of LC3 and phosphatidylethanolamine through RavZ, and can also block LAP induced by its infection. BopA, an important effector protein encoded by the TTSS3 gene of *B. pseudomallei*, inhibits LC3 recruitment. The high and low expression of VAMP3 in *Y. pseudotuberculosis* can localize in autophagic vesicles of monolayer and bilayer membranes, respectively. VAMP7 inhibits the maturation of YCVs and disrupts LAP and autophagy, but the mechanism remains unclear. The spread of *S. flexneri* is dependent on the function of T3SS. IcsB blocks the recruitment of LC3 by blocking the binding of the ATG5 to the surface protein VirG of *S. flexneri*. IcsB is also thought to recruit the actin-associated protein TOCA-1, promoting cell-to-cell spread. Likewise, VirA prevents LC3 recruitment to *S. flexneri*-containing vesicles. Specifically, the inactivation of Rab1 by VirA inhibits the effect of ER on GA transport, but it remains unclear how Rab1 is involved in LC3 recruitment to *S. flexneri*-containing vacuoles. *S. aureus* was found to establish an intracellular niche in neutrophils, the mechanism of which has not been elucidated. *S. pneumoniae*-induced LC3 recruitment is dependent on pneumolysin.

The LAPosome fuses with lysosomes to degrade bacteria, but bacteria have evolved survival mechanisms to prevent this fusion process. The main mechanisms include blocking or destroying the production of phagosomes and impairing the fusion of phagosomes with lysosomes. Each of these two main methods creates an ideal environment for bacteria to replicate and survive in the body. Different bacteria have evolved various strategies to deal with LAP-promoted killing. Several typical strategies of bacteria in dealing with LAP are discussed below (Figure 3, Table 1).

### 3.1. Legionella

#### 3.1.1. *Legionella dumoffii*

*Legionella dumoffii* (*L. dumoffii*) is a Gram-negative intracellular parasitic bacterium. After the infection of host cells, host phagocytosis mediates the entry of *L. dumoffii* into cells, and upon entry, most *L. dumoffii* bacteria settle in endoplasmic reticulum-like vacuoles and replicate within them. The bacterial Dot/Icm type IV secretion system (T4SS) is required for both *L. dumoffii* replication and the formation of the LAPosome. In mouse embryonic fibroblasts, LC3 binds to *L. dumoffii*-containing vacuoles (LdCVs) in a Dot/Icm T4SS-dependent manner, resulting in bacterial degradation. This process requires TLR2, Rubicon, and diacylglycerol (DAG) signals [29] and downstream NADPH oxidase, but not the mammalian target of rapamycin (mTOR)-ULK-1-like autophagy-activating kinase (ULK1/2) signaling pathway [21]. Mutation or deletion of Dot/Icm T4SS can affect the recruitment of LC3 to LdCVs and affect the maturation of the phagosome. Hubber’s team also found that TLR2 knockout mouse-derived macrophages, in wild-type mouse BMDM and non-phagocytic HEK cells, could enhance the level of LC3 recruitment by LdCVs and that TLR2 and ROS triggered LAP to play a role through the DAG-NADPH axis. In addition, plasma membrane damage did not affect LAP activation in LdCVs. LdCVs do not bind to selective autophagy-related molecules (such as ubiquitin or adaptor proteins), thereby precluding the effects of autophagy and P62-mediated selective autophagy. In summary, the survival of *L. dumoffii* in host cells is largely limited by LAP [29]. 

#### 3.1.2. *Legionella pneumophila*

*Legionella pneumophila* (*L. pneumophila*) is the main pathogen in the genus *Legionella* causing disease in humans and is mainly transmitted through aerosols. Like *L. dumoffii*, host phagocytosis mediates the entry of *L. pneumophila* into cells, and *L. pneumophila* evades killing through its Dot/Icm T4SS and the effector protein RavZ, thereby remodeling phagocytic vesicles into a replicative niche, which are usually called *L. pneumophila*-containing vacuoles (LpCVs) and effectively inhibit host autophagy [29,68,69]. *L. pneumophila* secretes more than 300 effectors into the host cytoplasm through its unique Dot/Icm Type-IVB secretion system, among which more than ten effectors, such as AnkB, SidC, LubX, SidH, LegU1, GobX, RavD, DupA, DupB, SidJ, Ceg23, MvcA, MavC, and SidE, are known to be involved in the regulation of host ubiquitination. *L. pneumophila* can evade lysosomal fusion, continue to carry out intracellular replication, and regulate host-cell apoptosis and other physiological activities [68,70,71]. THP-1 cells were infected with a *L. pneumophila* strain lacking RavZ or a wild-type *L. dumoffii*, which does not encode RavZ. It is concluded that RavZ has effect on LC3 recruitment to LpCVs and LdCVs.

RavZ has the conjugate function of irreversibly uncoupling LC3 from the phospholipid membrane [69] and can also block LAP induced by *L. pneumophila* infection [29], thereby increasing the survival rate of bacteria, which reflects the difference between *L. pneumophila* and *L. dumoffii*. In addition, even without the presence of RavZ, LpCVs will not recruit LC3 [29]. LpCVs are highly resistant to selective autophagy, suggesting that *L. pneumophila* has other mechanisms allowing it to evade autophagy [30,69].

### 3.2. Burkholderia pseudomallei

*Burkholderia pseudomallei* (*B. pseudomallei*) is a Gram-negative intracellular parasitic bacterium. The secretion system of bacteria plays an important role in the pathogenic process, such as the type III secretion system (T3SS) and the type VI secretion system (T6SS), which are the two most critical virulence-related factors in the intracellular survival of *B. pseudomallei* [31]. T3SS plays important roles in the processes by which this bacterium invades epithelial cells [32], secretes virulence factors, and evades host immunity [33,72]. Studies have shown that *B. pseudomallei* strains lacking the T3SS proteins BopA [33], BsaQ [73], BopE [74], and BipD [75] have a reduced ability to escape vesicles. For example, BopA may use the LC3-interacting region (LIR) sequence to inhibit LC3 recruitment to the pathogen-associated phagosome structure. The BopA mutant-infected RAW 264.7 macrophage cells show reduced virulence in mouse melioidosis models, suggesting that its role in intracellular survival is critical for the pathogenesis of *B. pseudomallei* [72]. It has been reported that BopA contains a Rho GTPase inactivation domain at its carboxy terminus, which may function as a protease or acyltransferase acting on host molecules [76]. Another report showed that BopA also contains a cholesterol-binding domain. Binding of cholesterol by BopA might lead to the accumulation of cholesterol on phagosome membranes, which could limit lysosomal recognition and fusion [77].

### 3.3. Listeria monocytogenes

*Listeria monocytogenes* (*L. monocytogenes*) is a typical intracellular parasitic bacterium. Studies have shown that the effect of LC3 on *L. monocytogenes* killing by macrophages is completed by LAP and is not associated with autophagy. Therefore, LAP is an important mechanism by which the body resists *L. monocytogenes* infection [26]. During *L. monocytogenes* infection of the host, LAP is closely related to mitochondrial Ca^2+^ signaling and the β_2_ integrin Mac-1.

#### 3.3.1. LAP and Mitochondrial Ca^2+^ Signal Transduction

In innate immune cells, mitochondria play an important role in pathogen defense [78]. In the process of the phagocytosis of bacteria, mitochondrial-derived vectors preferentially deliver ROS to phagosomes to kill bacteria [79]. At present, whether acetyl-coenzyme A (acetyl-CoA) accumulation in the junction region of mitochondria and phagosomes is the main factor modifying the Rubicon protein is not clear. The mitochondrial Ca^2+^ uniporter (MCU) is a highly selective Ca^2+^ channel [34,35,36,80]. The absence of MCU is conducive to the integrity of phagosomes and phagosome–lysosome fusion to promote the formation of LAP. *L. monocytogenes* promotes the uptake of mtCa^2+^ by regulating MCU to enhance the activity of pyruvate dehydrogenase (PDH), thereby inducing the production of acetyl-CoA. Acetyl-CoA acetylates Rubicon, which results in a decrease in Rubicon content and inhibition of the interaction between Rubicon and the NOX2 complex, thereby inhibiting the formation of LAP and promoting the survival of bacteria in host cells [81]. Therefore, *L. monocytogenes* regulation of MCU may be a strategy conducive to bacterial survival.

#### 3.3.2. LAP and β_2_ Integrin Mac-1

LAP caused by *L. monocytogenes* infection is derived from β_2_ integrin Mac-1 (CR3, integrin α_M_β_2_), which is a receptor that recognizes a variety of microbial ligands [26]. The interaction between *L. monocytogenes* and Mac-1 induces acid sphingomyelinase (ASMase)-mediated changes in membrane lipid composition and hydrolyses sphingomyelin to ceramide and phosphorylated choline. Ceramide-rich membrane receptors promote the assembly and activation of NOX2, and at the same time, peritoneal macrophages from WT and CD11b^−/−^, ASMase^−/−^, and NOX2^−/−^ mice were infected with WT, Δhly, or HK *L. monocytogenes.* To produce DAG accumulation on the phagosomes of *L. monocytogenes* to promote NOX2 activity [37,38], which is the process required to assemble NOX2 [82], NOX2-derived ROS can induce LC3 recruitment to phagosomes containing *L. monocytogenes* [21,37,83], promote the fusion of *L. monocytogenes*-containing phagosomes and lysosomes, and enhance the killing effect of bactericidal acid hydrolase on *L. monocytogenes*, thereby enhancing the resistance of macrophages to *L. monocytogenes*. Studies have also found that *L. monocytogenes* uses its own virulence factor 1-phosphatidylinositol phosphodiesterase A, B, (PLCA, B) and actin assembly-inducing protein A (ActA) to protect *L. monocytogenes* from targeted recognition, killing, and degradation by xenophagy. *L. monocytogenes* can escape killing by autophagy [26,84,85,86,87]; however, targeted killing by LAP cannot be avoided. Early studies proposed that *L. monocytogenes* can use LAP to establish a site that is conducive to *L. monocytogenes*’ survival and replication [37,87]. Subsequent studies found that this view was not valid, because the presence of virulence factors in *L. monocytogenes* that could evade killing by LAP has not yet been discovered. In addition to LAP, in recent years, a pore-forming toxin-induced noncanonical autophagy pathway (PINCA) has been reported to be induced by damage to the phagosome membrane by *L. monocytogenes* pore-forming toxin, i.e., listeriolysin O [88]. 

### 3.4. Streptococcus pneumoniae

*Streptococcus pneumoniae* (*S. pneumoniae*) is a Gram-positive coccus. Recent studies have found that after *S. pneumoniae* infection of mouse bone marrow-derived macrophages (BMDMs), the main killing mechanism of *S. pneumoniae* is ROS-mediated LAP rather than the direct bactericidal activity of ROS [17] and autophagy [39]. Studies have shown that the killing effect of LAP is different in BMDMs from mice of different ages after *S. pneumoniae* infection. The effect of LAP in mice at 2 months is better than that in mice at 20 to 22 months. LC3 conversion and recruitment were significantly reduced in BMDMs between 20 and 22 months of age, suggesting that the effect of LAP decreased with age [40]. Importantly, *S. pneumoniae*-induced LC3 recruitment is dependent on the pore-forming toxin pneumolysin (PLY) [39]. PLY is an important cytolytic toxin secreted by *S. pneumoniae* [41,42,43]; however, the role of PLY in LAP defense in mice of different ages has not been explored. To determine whether this killing was mediated by LAP, the authors evaluated *S. pneumoniae* killing in BMDMs lacking ATG14, ATG7, Rubicon, or NOX2. The association of LC3 with *S. pneumoniae*-containing phagosomes required components specific for LAP, such as Rubicon and NOX2.

### 3.5. Mycobacteria

#### 3.5.1. *Mycobacterium tuberculosis*

*Mycobacterium tuberculosis* (*M. tuberculosis*) is a pathogen that causes tuberculosis and mainly parasitizes host macrophages [89,90,91]. Studies have shown that to improve the survival rate of *M. tuberculosis* in host cells, *M. tuberculosis* has evolved a variety of strategies to prevent the occurrence of autophagy in host cells [44,92,93]. These strategies enable *M. tuberculosis* to evade autophagy [44,45,46,47,48]. Recent studies have shown that the ability of *M. tuberculosis* strains H37Rv and ΔCpsA in BMDMs and human THP-1 cells require CpsA to survive in macrophages. RAW264.7 cells transfected with CpsA or control mRNA were infected with H37Rv and ΔCpsA. It is concluded that CpsA is sufficient to inhibit LAP. CpsA contains the LytR-CpsA-Psr (LCP) domain and acts upstream of NOX2 [21,83]. It blocks the recruitment of NOX2 to phagosomes [27,49] to evade LAP killing.

#### 3.5.2. *Mycobacterium marinum*

*Mycobacterium marinum* (*M. marinum*) is a bacterium existing in seawater and freshwater. For *M. marinum*, LC3 recruitment depends on the viability of the bacteria and the type VII secretion system [50]. The type VII secretion system may secrete a pore-forming protein into the vacuole containing *M. marinum*, thereby destroying the integrity of the vacuole membrane and allowing *M. marinum* to escape to the host cytoplasm [94]. CpsA is required for the cell wall integrity and virulence of *M. marinum*. Raw264.7 macrophages (Raw cells) were infected with *M. marinum* in a zebrafish model, and CpsA deficiency was found to weaken the virulence of *M. marinum* [51,52,53,54]. In summary, we speculate that CpsA may be an effective drug target that can promote LC3 transport by blocking CpsA in host-targeted therapy.

### 3.6. Shigella flexneri

*Shigella flexneri* (*S. flexneri*) is an intestinal pathogenic bacterium. After *S. flexneri* infects colonic epithelial cells, it forms membrane protrusions that protrude into adjacent cells and decompose into double-membrane vacuoles (DMVs), thereby spreading between cells. LC3 is recruited into DMVs, and *S. flexneri* is targeted by LC3, which is the defense mechanism of host cells against intracellular pathogens [55], thereby promoting the clearance of pathogens by host cells. However, studies have found that the cholesterol-binding domain of the effector IcsB secreted by the T3SS in the early stage of *S. flexneri* infection is related to the escape of *S. flexneri* from LC3-containing vesicles [56]. WT or ΔIcsB *S. flexneri* infection of HeLa cells confirmed that the defect in the host protein transducer of Cdc42-dependent actin assembly (Toca-1) recruitment observed for the deletion strain was due to the absence of IcsB. 

At the late stage of infection (4–6 hours), IcsB is required for *S. flexneri* to escape autophagy [56,57]. IcsB blocks LC3 recruitment by blocking the binding of the ATG5 to the *S. flexneri* surface protein VirG, allowing bacteria to escape from host cells at the late stage of infection [94,95] and from liposomes during secondary infection, thereby achieving bacterial spread between cells [55,56,96,97]. The role of IcsB in bacterial escape has been controversial in recent years. Relevant studies have shown that the role of IcsB is not to attenuate the recruitment of DMVs to LC3. Notably, a study found that a small number of bacteria showed delayed escape from the cell vacuole, but still successfully escaped, indicating that an IcsB-independent mechanism also facilitates the escape of DMVs [55]. The immunomodulatory role of relevant effectors in different cell types may be a topic of major interest in the next few years.

### 3.7. Yersinia pseudotuberculosis

In recent years, studies have shown that the autophagy mechanism triggered by *Yersinia pseudotuberculosis* (*Y. pseudotuberculosis*) is not related to the T3SS [58]. In the early stage of *Y. pseudotuberculosis* invasion in epithelial cells, *Y. pseudotuberculosis* replicates inside LC3-positive vacuoles [59,60]. Vesicle-associated membrane protein 3 (VAMP3) is involved in the process of bacterial replication in LC3-containing vesicles. In addition, vesicle-associated membrane protein 7 (VAMP7) plays a role in the recruitment of LC3. For example, following infection of HeLa cells, EGFP-VAMP3 and GFP-VAMP7 co-distributed with *Y. pseudotuberculosis*. These results reveal that VAMP3 and VAMP7 are sequentially recruited into YCVs to impair the maturation of YCVs, which is conducive to the proliferation of bacteria in host cells [58]. Studies have found that *Y. pseudotuberculosis* provides a replication site for itself by impeding LAP and autophagy, thereby allowing it to survive in mouse BMDMs [59]. Studies published to date have not yet shown how the body selects LAP and autophagy.

### 3.8. Salmonella Typhimurium

*Salmonella Typhimurium* (*S. typhimurium*) is a Gram-negative bacillus, and the multiple virulence factors of *S. typhimurium* are the main reason for its survival, reproduction, and spread in the host. Ibarra and Van et al. reported the function of virulence factors in invading host cells and replicating in cells [98,99]. Mesquita et al. initially explored the role of virulence factors in host autophagy [100]. In recent years, Masud et al. confirmed the defense function of LAP in the host in zebrafish biological models. In a zebrafish embryonic infection model, macrophages were more important for *S. typhimurium* defense than neutrophils. Moreover, knockout of ATG5 significantly increased the survival rate of *S. typhimurium* in the host [9,83,101,102]. However, knockout of ATG13 did not play a significant role in host defense against *S. typhimurium* infection. Therefore, LAP could be concluded to be the main mechanism of the host defense against *S. typhimurium* infection. Finally, wild-type and virulence factor mutant strains in *S. typhimurium* were used to infect zebrafish embryos for an experiment, showing that *S. typhimurium* strains carrying mutations in virulence factors PhoP, PurA, SipB, SsrB, and FlhD are all able to trigger LAP, and that all mutants except the SsrB-deficient strain become more virulent in a LAP- deficient host [61,65]. *S. typhimurium*-infected WT and ATG5, ATG13, and Rubicon knockdown, were shown to be required for LC3 recruitment and for the successful clearance of bacteria in the zebrafish model, providing in vivo evidence for the anti-*Salmonella* function of LAP.

Studies have shown that the advantages of *S. typhimurium* in pathogenicity and invasiveness are mainly reflected in the role played by many virulence factors, including *Salmonella* pathogenicity islands (SPIs), *Salmonella* plasmid virulence (*spv*), and virulence factors. As virulence factors are closely related to pathogenicity [99], the molecular structure of T3SSs encoded by SPIs is the main cause of pathogenicity [103]. *spv* can inhibit the formation of autophagosomes, the response of type I interferon, and phagocyte recruitment and play an important role in intracellular reproduction and the evasion of immune system clearance [62,63,64]. Among the virulence factors of *S. typhimurium*, flagellar transcriptional activators (*FlhD*) can regulate the expression of flagellin and promote the secretion of cytokines by the immune system, which is conducive to the defense of LAP in the zebrafish host. However, mutations in *FlhD* resulted in reduced LC3 recruitment and increased *S. typhimurium* virulence [61,65]. *PhoP* and *PurA* promote *S. typhimurium* replication in zebrafish hosts, while the loss of *PhoP* and *PurA* virulence factors increases LC3 recruitment, and LAP enables zebrafish hosts to clear *S. typhimurium* more quickly. In addition, *S. typhimurium* has an SCV structure, which can deubiquitinate ubiquitinated aggregates and polyubiquitinated proteins surrounding the SCV through the virulence factor SseL, thereby inhibiting the P62-mediated selective autophagy reaction, which is conducive to survival in host cells [100,104]. The above studies indicate that virulence factors are an effective escape method and further provide a basis for LAP as a host protection mechanism for macrophages.

### 3.9. Staphylococcus aureus

*Staphylococcus aureus* (*S. aureus*) is a Gram-positive bacterium that is widely present in the natural environment and is considered an extracellular pathogen. However, an increasing number of studies have shown that *S. aureus* can partially survive in phagocytes [105]. Studies have shown that autophagy and LAP play an antagonistic role in *S. aureus*-infected neutrophils in zebrafish larvae, and *S. aureus* uses LAPosomes to escape killing by autophagy. Prior studies found that after LAP-mediated phagocytosis of *S. aureus* by neutrophils, *S. aureus*-containing nonacidic phagosomes proliferated and disseminated [66]. They also reported that the use of *S. aureus* to infect transgenic zebrafish larvae revealed a difference in responses between phagocytes. First, in the process of LC3 modification of *S. aureus* phagocytic macrophages and neutrophils, the modification effect of LC3 in neutrophils was delayed compared to that of macrophages [66]. Moreover, *S. aureus*-infected WT and irf8, cyba/p22^phox^, sqstm1, ATG5, and ATG16L knockdown was observed in a zebrafish embryo model. As observed in the late stage of infection, more LAPosomes were formed by neutrophils than macrophages after phagocytosis of *S. aureus*. These results indicate that neutrophils may inhibit autophagic flux [105]. In addition, Lv et al. showed that *S. aureus*-induced autophagy contributed to the phagocytosis of macrophages. Second, NOX activity was inhibited by knocking out the membrane-bound subunit cyba/p22^phox^ of NOX. Knockout of cyba/p22^phox^ had no effect on the survival of *S. aureus* in macrophages in zebrafish larvae, while the effect of LC3 on the modification of *S. aureus*-containing neutrophils was significantly reduced, and the resistance of zebrafish larvae to *S. aureus* infection was increased. These results indicate that NOX-mediated *S. aureus* did not play an important role in the processing of bacteria in zebrafish macrophages. Studies have shown that sequestosome 1 (Sqstm1) is an important selective autophagy adaptor protein and an aptamer protein in the ubiquitination system that regulates intracellular protein degradation, and mainly plays a role in the protection of neutrophils in *S. aureus*-infected zebrafish larvae [106,107]. Knockdown of Sqstm1 showed that the LAPosome formed in macrophages and neutrophils was not affected by Sqstm1, and LC3 recruitment did not require live bacteria, indicating that the autophagy reaction caused by membrane damage was excluded [66]. Second, LAPosome formation requires ROS produced by NOX2, which is another hallmark of LAP [21,83]. In summary, the LC3-mediated response in *S. aureus* was further confirmed to be LAP [48,65,66].

In summary, in a zebrafish model, after the LAP-mediated phagocytosis of *S. aureus* by LC3 on neutrophils, *S. aureus*-containing nonacidified phagosomes are formed [67,108,109,110], which may serve as an intracellular niche that promotes disease development. The occurrence of this process is harmful to the host and can attenuate LAP by inhibiting the activity of NOX [21,37,83], thereby increasing the resistance of zebrafish larvae to *S. aureus*.

## 4. Potential Therapeutic Strategies

With an understanding of the antibacterial function of LAP and the mechanisms by which bacteria escape LAP, researchers can design therapeutic strategies based on each step of the LAP process.

Bacterial recognition by receptors is the first step of LAP. TLRs are critical in host defense against pathogens by their ability to detect microorganisms and initiate immune responses by recognizing the pathogen-associated molecular patterns of bacteria. TLR4 is known to recognize lipids, glycoproteins, secreted proteins, and other surface ligands of *M. tuberculosis*, leading to rapid phagocytosis of the pathogen [111]. In addition, initiation of the LAP response in *L. dumoffii* requires pathogen recognition through TLR2 and diacylglycerol signaling. *B. pseudomallei* is recognized by TLR2 and TLR4 [112]. Earlier studies of *Salmonella* infection in mouse macrophages and human epithelial cells have shown that LAP may play a key role in the immune response, as triggering of TLR or FcγR induces LC3 in a ROS-dependent manner on the phagosome [83]. Mutation of the FlhD gene of *S. typhimurium* increases the high virulence of *S. typhimurium* in zebrafish and reduces the recruitment of GFP-LC3, which may be that the induction of LAP is dependent on the recognition of flagellin by TLR5, but TLR5 has not yet been identified in the LAP-signaling pathway. In conclusion, the in-depth study of the role of TLRs ligand–receptor interaction in LAP can provide a basis for the application of receptor agonists or inhibitors.

**Figure 4:**
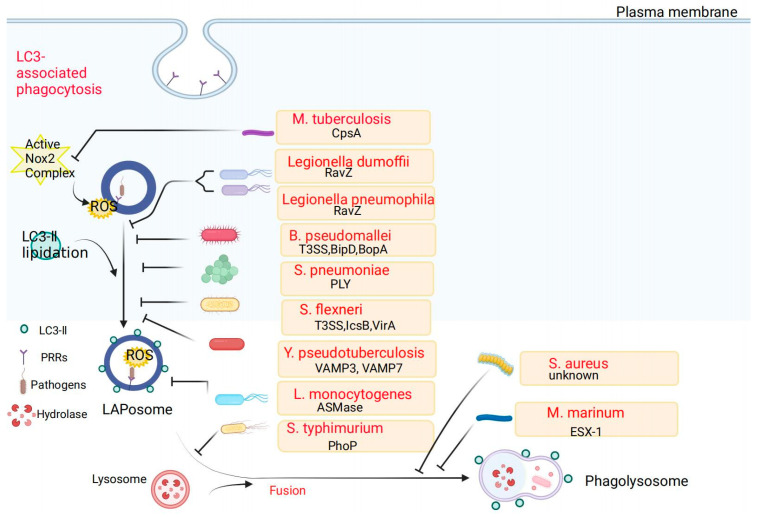
Strategies of bacteria to evade LAP. Bacteria have evolved strategies to interfere with LAP formation, such as NADPH oxidase formation, LC3 lipidation, or fusion with lysosomes. *L. dumoffii* and *L. pneumophila* escape LAP via the T4SS effector protein RavZ, making it unable to lipidate LC3 and phagosomes to fuse with lysosomes into endoplasmic reticulum-like vacuoles. *B. pseudomallei* uses T3SS and its effector protein BipD and transporter BopA to evade the killing of LAP. *L. monocytogenes* upregulates mitochondrial calcium signaling to acetylate Rubicon and inhibit the interaction of Rubicon with the NOX2 complex, thereby inhibiting LAP formation. *S. pneumoniae* triggers the PLY-dependent lipidation of LC3, which is recruited to internalized bacteria. *M. tuberculosis* can secrete the CpsA protein, which prevents NOX2 from assembling on phagosomes, thereby inhibiting ROS production and evading the killing of LAP. *M. marinum* infection is circumvented by a lack of lysosomal acidification and a lack of lysosomal proteases. *S. flexneri* detaches from the LAPosome under the action of its T3SS and its effector proteins. On the other hand, IcsB secreted by T3SS can recruit the host protein Toca-1 to the surrounding intracellular bacteria. The interaction of these two proteins can inhibit the early phagosome LC3 recruitment in cells. VAMP3 and VAMP7 consecutively colocalize with *Y. pseudotuberculosis*-containing vacuoles. The PhoP regulatory protein of *S. typhimurium* reduces TLR activation and plays a role in inhibiting phagolysosomal fusion. After *S. aureus* is internalized by neutrophils, the absence of the acidification of LAPosomes provides a replicative niche for *S. aureus*.

Once invading the cell, the bacteria interfere with important stages of the LAP process for their survival (Figure 4). During the formation of NADPH oxidase, *M. tuberculosis* can secrete the CpsA protein, which prevents NOX2 assembly on phagosomes and then avoids the killing effect of LAP through inhibiting ROS production. It is not clear whether the NADPH oxidase assembly and activation can be effectively activated by adding NADPH oxidase activators, thus achieving the efficient clearance of bacteria. In the process of LC3 recruitment and LC3 lipidation, bacteria, such as *Legionella*, *B. pseudomallei*, *S. pneumoniae, S. flexneri*, or *Y. pseudotuberculosis*, use their respective virulence factors to interfere with the recruitment of LC3 and inhibit LC3 lipidation to escape LAP killing. In the future, it is necessary to develop small-molecule drugs that selectively target bacteria-containing phagosomes to increase the recruitment capacity of LC3 on the surface of these phagosomes. In addition, the supplementation of specific autophagy-related proteins (such as ATG5, ATG12, ATG16) can rescue autophagy-related proteins that are competitively blocked by bacterially secreted virulence factors. Finally, during the fusion of phagolysosomes, *M. marinum* infection causes cells to lose the lysosomal acidification environment or reduce lysosomal protease, and *S. aureus* was found to establish a nonacidified intracellular niche for replication in neutrophils. Thus, novel therapeutic strategies may be made to kill bacteria that colonize in cells and that escape into the cytoplasm by increasing pH in lysosomes, or enhance the co-localization of LC3 or lysosomal proteins (such as LAMP1) with bacteria.

## 5. Conclusions and Perspectives

As a form of noncanonical autophagy, LAP enhances pathogen degradation by promoting phagosome–lysosome fusion and plays an immunomodulatory role in antimicrobial immunity. In recent years, different species of bacteria have been reported to be associated with LAP. LAP can efficiently remove invading bacteria and play an important defense role in the host. However, as shown in this review, in the long-term “arms race” with the host, bacteria have evolved multiple strategies to interfere with the LAP pathway to enhance infection efficiency and evade immune surveillance. Therefore, revealing the antibacterial mechanism of LAP and exploring the strategies of how bacteria evade or utilize LAP have become the main areas of focus for research.

The generation of ROS within the phagosome lumen depends on Rubicon activity, which is essential for LAP maturation. Most bacteria targeted by LAP have evolved ways of interfering with NOX2. For example, *L. monocytogenes* inhibits the occurrence of LAP by regulating MCU in the mitochondrial pathway. In fact, it affects Rubicon in the formation of LAP, which eventually leads to impaired NOX2 assembly and activation. Another example is that *M. tuberculosis* evades the killing of LAP by blocking the recruitment of NOX2 to the phagosome. In addition to oxidative activity against bacteria, ROS are also required to recruit downstream LAP components such as ATG7 and LC3. Studies have shown that ROS generated by NOX2 stabilize the LAPosome by inhibiting deconjugation from the LAPosome cytosolic surface. NOX2 resides on the LAPosome membrane and generates ROS to oxidatively inactivate the protease ATG4B, which releases LC3B from the LAPosome.

The T3SS effector proteins BopA and IcsB are essential for the ability of *B. pseudomallei* and *S. flexneri*, respectively, to evade vesicles for intracellular survival, possibly because the effector proteins BopA and IcsB contain cholesterol-binding domains. As a result, cholesterol accumulates on the phagosome membrane after binding with the effector proteins, which affects the normal function of the phagosome and subsequent physiological processes. The T3SS effector protein BopE of *B. pseudomallei* can induce the rearrangement of actin in host cells by catalyzing the Rho GTPase of host cells, thereby facilitating bacterial invasion. Meanwhile, T3SS is also required for the spread of *S. typhimurium* and *S. flexneri* in host cells. In fact, both bacteria force themselves into epithelial cells by manipulating the actin cytoskeleton. Additionally, *Shigella* shares a mechanism for invading host cells with certain T3SS-expressing bacteria, such as *Salmonella enterica*. T4SS is also an important virulence factor for a variety of bacteria, is widely distributed in Gram-negative bacilli and Gram-positive cocci, encodes a variety of structural and functional pilus proteins, and plays an important role in the pathogenic process of bacteria. As mentioned in this review, T4SS plays an integral role in the ability of *L. dumoffii*, *L. pneumophila*, and *M. marinum* to escape lysosomal fusion and survive in cells.

The mode of action of LAP may depend on the cell type. Like mouse BMDMs, LAP promotes the fusion of the LAPosome with lysosomes to accelerate pathogen clearance, while in mouse plasmacytoid dendritic cells (pDCs), LAP redirects endocytic cargo to cells containing endosomes of TLR9. This finding also raises the question of how bacteria choose innate immune pathways. It is well known that PRRs include transmembrane receptors and cytoplasmic receptors, but the difference between PRRs in phagosomes and LAPosomes and how to further trigger downstream signaling cascades to clear bacteria have not been clarified. Therefore, further elucidation of the way in which PRRs are involved in LAP will open a new research field for immunity against bacterial infections.

In addition, most of the current research on LAP focuses on macrophages. As the most abundant white blood cells in the human circulatory system, neutrophils are also the most common innate immune cells. They are the first to reach the site of inflammation and are mainly responsible for host defense, immune regulation, and tissue damage repair. They play a role in the process of antibacterial infection. The role of neutrophils in LAP has been less commonly reported in recent studies. Is LAP selective to phagocytes in the process of antibacterial infection? Does LAP need to coordinate among various phagocytes, so that multiple phagocytes antagonize or cooperate with each other to mediate the immune response of the body? Fully exploring these issues may be beneficial for understanding the pathogenesis of related diseases. 

## Figures and Tables

**Table 1:** Bacterial pathogens and their evasion strategies.

Bacteria	Virulence Factor	LAP-Specific Evasion Mechanisms	References
*Legionella dumoffii*	RavZ	Evasion of LAP by the T4SS effector protein RavZ, which inhibits LC3 lipidation and phagosome-lysosome fusion	[21,29]
*Legionella pneumophila*	RavZ	Evasion of LAP by the T4SS effector protein RavZ, which inhibits LC3 lipidation and phagosome–lysosome fusion	[29,30]
*Burkholderia pseudomallei*	BopA, BipD	Escapes from LAPosome and inhibits the recruitment of LC3 via BopA and BipD	[31,32,33]
*Listeria monocytogenes*	LLO	Upregulation of mitochondrial calcium signaling leads to the acetylation of Rubicon, interfering with LAP formation and the recruitment of NADPH oxidase	[26,27,34,35,36,37,38]
*Streptococcus pneumoniae*	PLY	Unknown	[39,40,41,42,43]
*Mycobacterium tuberculosis*	CpsA	Evasion of LAP by secreting CpsA protein to prevent NOX2 from assembling on phagosomes and inhibiting ROS production	[27,44,45,46,47,48,49]
*Mycobacterium marinum*	Type VII secretion systemESX1, CpsA	Evasion of LAP via non-acidifying LC3-positive vesicle, which is established through the ESX-1 secretion system	[50,51,52,53,54]
*Shigella flexneri*	IcsB, VirA	Evasion of LAPosome by inhibition of LC3 recruitment through interaction between TOCA-1 and IcsB	[55,56,57]
*Yersinia pseudotuberculosis*	Unknown	VAMP3 and VAMP7 co-localize with YCVs resulted in inhibiting LC3 recruitment	[58,59,60]
*Salmonella enterica Typhimurium*	PhoP, PurA, FlhD	Reduce TLR activation, inhibit LC3 recruitment, and inhibit phagolysosomal fusion	[61,62,63,64]
*Staphylococcus aureus*	Unknown	Forming spacious GFP-LC3-positive vacuoles that do not acidify	[48,65,66,67]

## Data Availability

Not applicable.
